# Synergy and Order Effects of Antibiotics and Phages in Killing *Pseudomonas aeruginosa* Biofilms

**DOI:** 10.1371/journal.pone.0168615

**Published:** 2017-01-11

**Authors:** Waqas Nasir Chaudhry, Jeniffer Concepción-Acevedo, Taehyun Park, Saadia Andleeb, James J. Bull, Bruce R. Levin

**Affiliations:** 1 National University of Sciences and Technology, Islamabad, Pakistan; 2 Department of Biology, Emory University, Atlanta, Georgia, United States of America; 3 Department of Molecular Biosciences, The University of Texas at Austin, Austin, Texas, United States of America; 4 Department of Integrative Biology, The University of Texas at Austin, Austin, Texas, United States of America; Universiteit Leiden, NETHERLANDS

## Abstract

In contrast to planktonic cells, bacteria imbedded biofilms are notoriously refractory to treatment by antibiotics or bacteriophage (phage) used alone. Given that the mechanisms of killing differ profoundly between drugs and phages, an obvious question is whether killing is improved by combining antibiotic and phage therapy. However, this question has only recently begun to be explored. Here, *in vitro* biofilm populations of *Pseudomonas aeruginosa* PA14 were treated singly and with combinations of two phages and bactericidal antibiotics of five classes. By themselves, phages and drugs commonly had only modest effects in killing the bacteria. However some phage-drug combinations reduced bacterial densities to well below that of the best single treatment; in some cases, bacterial densities were reduced even below the level expected if both agents killed independently of each other (synergy). Furthermore, there was a profound order effect in some cases: treatment with phages before drugs achieved maximum killing. Combined treatment was particularly effective in killing in *Pseudomonas* biofilms grown on layers of cultured epithelial cells. Phages were also capable of limiting the extent to which minority populations of bacteria resistant to the treating antibiotic ascend. The potential of combined antibiotic and phage treatment of biofilm infections is discussed as a realistic way to evaluate and establish the use of bacteriophage for the treatment of humans.

## Introduction

The increasing incidence of multi-drug resistant pathogens and the virtually dry pipeline of new antibiotics has been described as a “perfect storm” in public health [1]. While the apocalyptic pronouncements of an end of the antibiotic era may be overstating the case, it is clear that inherited resistance is a major clinical and public health problem [2]. Bacterial infections that were readily treated in the past are now difficult to treat because the pathogens are resistant to the antibiotics previously employed [3]. In some cases, they are virtually untreatable, like the carbapenem-resistant Enterobacteriaceae [4] and the recently discovered colistin resistant *E*. *coli* encoding mcr-1 [5].

Inherited resistance is not the only reason antibiotic treatment fails. Even when the pathogen responsible for an infection is fully susceptible to the treating antibiotic, it may be phenotypically refractory to the drug for a number of reasons, perhaps the most prominent of which is the physical structure of its populations [6]. In the world beyond the laboratory, bacteria rarely exist as planktonic cells in liquid, but rather reside as colonies or micro-colonies on surfaces or semi-solids and commonly imbedded in polysaccharide matrices known as biofilms [7]. Bacteria within biofilms are more refractory to antibiotics than they are as planktonic cells [8–10].

How does one deal with the increasing frequency of pathogens that are genetically resistant to multiple antibiotics and phenotypically resistant because of the physical structure of their population? One answer to this question brings us back a long past era and a therapy that has been virtually eclipsed by antibiotics, bacteriophage (phage) therapy. While there are clear limitations to the use of phage as the sole agent for treating bacterial infections [11, 12], it has been proposed that these bacterial viruses may be an effective adjunct to antibiotic treatment [13–15], and there is evidence in support of this proposition [16]. It has also been suggested that for ecological and physiological reasons, bacteriophage are likely to be more effective than antibiotics in killing bacteria within biofilms: (i) The polysaccharide depolymerase enzymes produced by phage are capable of breaking down the extracellular matrix of biofilms; antibiotics are not. (ii) By lysing the bacteria in the exterior of biofilms, lytic phages expose cells within these structures to exogenous nutrients and thereby make the cells in the interior of the biofilm more metabolically active and thus more susceptible to killing by antibiotics [17–19].

*Pseudomonas aeruginosa* is a particularly appealing candidate for combination phage and antibiotic therapy. In addition to being the immediate cause of mortality of many cystic fibrosis patients [20], *P*. *aeruginosa* is a major source of morbidity and mortality in burn patients [21], immune-compromised patients [22] and patients with the skin ulcers that commonly plague diabetics [23]. *P*. *aeruginosa* is naturally resistant to many antibiotics and has evolved resistance to many others [24]. There is, however, an abundance of *P*. *aeruginosa* phages that can infect and kill these bacteria and that can be isolated from a variety of sources, including sewage [25–27].

A number of studies have shown lytic phage to be effective in reducing the densities of bacteria in experimental infections with laboratory mice [28] and in some cases being more effective than antibiotics in the preventing mortality due to these infections [11, 29]. A few studies demonstrated the efficacy of phage and antibiotic combinations on planktonic cultures of *P*. *aeruginosa* [14–16] and biofilms [30]. Chan and colleagues observed what might be called ‘evolutionary’ synergy between antibiotics and phage [31]: resistance to a phage that uses an outer membrane porin as a receptor site led to increases in the susceptibility to antibiotics of different classes because resistance engendered a modification of the efflux pump responsible for resistance to these drugs. In short, the combination of drug and phage reciprocally blocked both pathways of resistance evolution.

In this investigation, we provide additional support for the clinical potential of using combinations of antibiotics and phage to treat biofilm infections with *P*. *aeruginosa*. Using two recently isolated lytic phages and *P*. *aeruginosa* PA14, we demonstrate that these viruses can increase the efficacy of several antibiotics commonly employed for treating *P*. *aeruginosa* biofilms. We also consider the effect of phages in limiting the ascent of minority populations resistant to the treating antibiotic.

### Terminology and concepts: Synergy and facilitation

A major emphasis in this paper is whether the combination of multiple agents improves killing. The motivation for such a focus is perhaps obvious; we want to know how best to kill bacteria. It is less obvious, however, that there are different categories of combined benefit, and some types of combined benefit may even work better than would be predicted from the separate effects. Not only may these superior interactions allow us to kill maximally, but they may also give us insight to additional ways of controlling bacteria that would never be discovered from working with single agents. We thus explain and define the nature of these interactions before proceeding to the empirical work.

**Fig 1:**
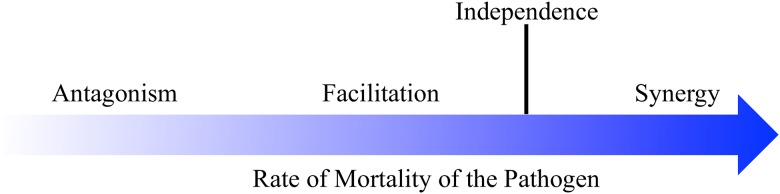
Possible interactions between two treatments on the rate of mortality of a target pathogen. Antagonism—in combination, the two kill the pathogen at a lower rate than the best of the treatments alone. Facilitation—together, the treatments kill at a rate greater than the best of the treatments alone but less than that if the treatments were acting independently, where they would kill at a rate equal to the product of the rates at which they act alone. Synergy—the pathogen is killed at a greater rate than that when the treatments are acting independently.

There is an extensive literature on the effects of combined treatment and a terminology to describe that effect [32–34]. In keeping with that literature, we use the word ‘synergy’ to indicate an outcome in which combined treatment kills a greater fraction of the bacteria than expected if the agents were acting independently (this independence is known as Bliss independence) [32]. There is a second, lesser zone of combined treatment benefit that is also of interest, that in which combined treatment is better than the best of the single treatments but is no better than if the drugs were acting independently. Since the literature is not united on what term to use for this latter concept, we refer to it as ‘facilitation’. Antagonism operates when combined treatment is worse than the best single treatment (Fig 1).

## Materials and Methods

### Culture and sampling media for the bacteria and phage

Liquid cultures of bacteria or bacteria and phage were grown in Lysogeny Broth (LB) (Difco Ref. #244620) supplemented with 2 mM CaCl_2_. Bacteria and phage densities were estimated by serial dilution in 0.85% saline. The bacteria were plated directly on LB hard (1.6%) agar. To estimate the densities of phage, serially diluted suspensions of these viruses were mixed with 0.1 mL of overnight cultures of ancestral *P*. *aeruginosa* PA14 to which 3 ml of LB soft (0.65%) agar was added and poured on the semi-hard (1%) agar plates.

### Bacteria and Phage

Joanna Goldberg generously provided the *P*. *aeruginosa* PA14 used in this study, designated as PA14. Ciprofloxacin and gentamicin resistant mutants of PA14, PA14Cip-R and PA14Gen-R were generated by serial passage of *P*. *aeruginosa* PA14 in the presence of increasing concentrations of gentamicin and ciprofloxacin [35]. The colonies selected for our experiments had minimum inhibitory concentrations (MIC) of 10μg/mL and 50 μg/mL for ciprofloxacin and gentamicin, respectively.

We isolated the *Pseudomonas* phage, designated NP1 and NP3, from a local sewage treatment plant (Atlanta GA). For this, we added LB broth with *P*. *aeruginosa* PA14 to a sample of the sewage suspension to enrich for phages that are specific to these bacteria and isolated single plaques on *P*. *aeruginosa* PA14 lawn. Cross-resistance was tested by spotting 100 μL of the high density of phages (10^9^ PFU/mL) on the soft agar lawns of the *P*. *aeruginosa* PA14. Clear zones were scored as sensitive. Phage NP1 formed zones on lawns PA14 cells that were resistant to NP3 and NP3 formed zones on lawns resistant to NP1. While wild-type *P*. *aeruginosa* PA14 is sensitive to both NP1 and NP3, the wild type *P*. *aeruginosa* PAO1 is sensitive to NP3 but resistant to NP1. Additional evidence for these phage having different receptor sites comes from experiments with transposon-mediated insertion sequence—mediated mutagenesis into potential receptors. When the AlgC gene of *P*. *aeruginosa* PAO1 is knocked out by transposon mutagenesis, this strain becomes resistant to both NP1 and NP3. The Δ AlgC mutant produces a truncated lipopolysaccharide core and lacks a common antigen indicating that these bacterial surface structures might entail the host receptor for NP3 phage attachment [36]. See the supporting information (S1 Text File) for electron-micrographic images of NP1 and NP3 phage (S1 Fig) and their respective DNA sequences (S2 Fig). The complete genome of phage NP1 and NP3 have been deposited in GenBank under accession number KX129925 and KU198331, respectively.

### Antibiotics

Bactericidal antibiotics of five classes were selected for use here: ceftazidime (Sigma), ciprofloxacin (AppliChem), colistin (Sigma), gentamicin (Sigma) and tobramycin (Tocris). All these drugs are commonly employed to treat infection with *P*. *aeruginosa* [37].

### Minimum inhibitory concentration

The MICs of *P*. *aeruginosa* PA14 to different antibiotics were estimated using micro dilution procedure [38, 39]. The estimated MICs of these drugs for *P*. *aeruginosa* PA14 are respectively ceftazidime 3.125, ciprofloxacin 0.8, colistin 2.5, gentamicin 6.25 and tobramycin 1.25 μg/mL.

### Epithelial cell cultures

Human epithelial nasopharyngeal Detroit 562 (ATCC^®^ CCl-138^™^) cell cultures were grown by the method of [40]. These epithelial cells populations were established in sterile 24-well, flat-bottom polystyrene tissue culture plates (Corning^®^ Costar^®^) at a concentration of 10^6^ cells/well and grown and maintained in Minimal Essential Medium (MEM) (GIBCO) containing 10% fetal bovine serum (Invitrogen GIBCO). These somatic cell cultures were incubated at 37°C and 5% CO_2_ (Nuare ^™^ US Auto flow CO_2_ water jacketed Incubator) for 8 to 10 days until cells formed a confluent monolayer with tight junctions. The medium was changed every 2 or 3 days until confluent growth was achieved.

### Biofilm preparation and assays

**Fig 2:**
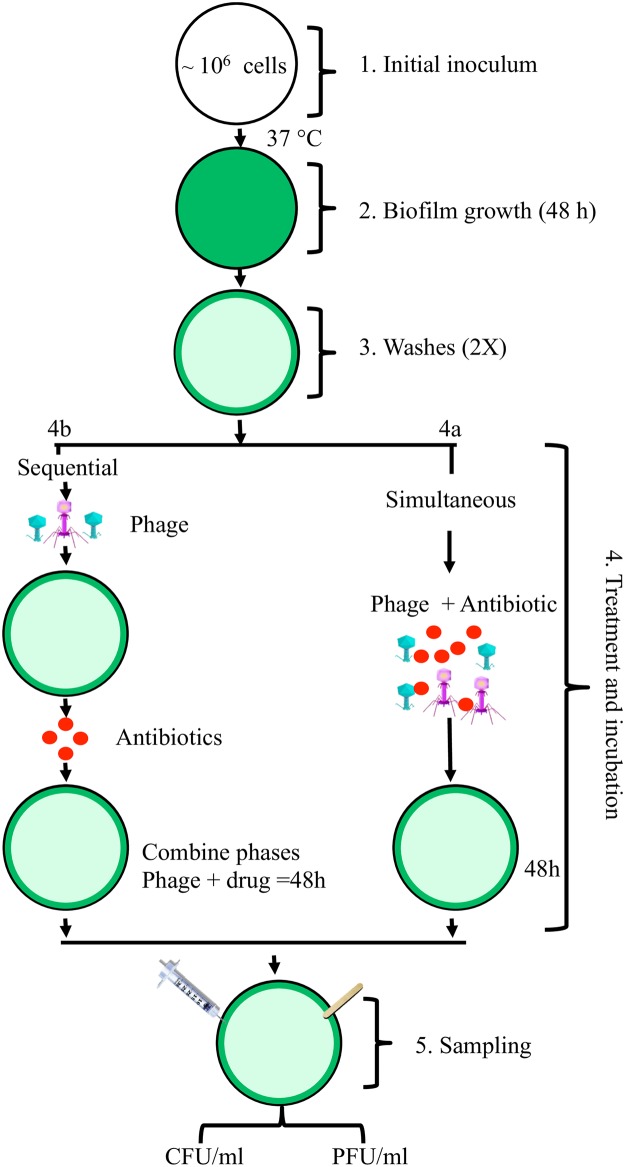
Schematic representation of the in-vitro procedure used to quantitatively explore the efficacy of antibiotics and phage for treating biofilm populations of bacteria. (1) The biofilms were established by inoculating 1 X10^6^ cells/mL in 2 mL LB in 24 well polystyrene plates. (2) These cultures were incubated for 48 hours without shaking to establish the biofilm. (3) The planktonic cells were removed with aspirator and wells were washed twice with saline. (4) These biofilm cultures were treated in one of two ways, (4a) Fresh LB with the phage (10^6^ PFU/mL and antibiotics were simultaneously added to the wells. (4b) Fresh LB with the phage mixture (10^6^ PFU/mL) was added first and then antibiotics were added with a delay of 4 or 24 hours. These cultures were incubated for total 48 hours starting from the addition of phages. (5) Following treatment, the cells were rendered planktonic by scraping the biofilm from walls of the well with the wooden applicator and later forced through a syringe needle to homogenize the cultures. The densities of viable bacteria and of phage were estimated by serial dilution and plating.

The protocol for these experiments is illustrated in Fig 2. *P*. *aeruginosa* PA14 biofilms were formed on plastic surfaces and on layers of epithelial cells in 24 well polystyrene plates. For the former, 2mL of LB with ~10^6^ cells from an overnight culture of the *P*. *aeruginosa* PA14 added into each well of 24 well macro-titer plates and incubated at 37°C without shaking for 48 hours to establish a mature biofilm. Our choice of mature biofilms was based on the assumption that symptomatic skin infections would be well established before treatment would be administered. Our criteria for "mature” was based on the fact that cell densities were no longer increasing at 48 hours as they were 8 and 24 hours, and that increased biofilm matrix was observed relative to 24 hours.

After incubation for 48 hours in the absence of treatment, the liquid from the cultures was removed with an aspirator and the wells washed twice with 0.85% saline to remove the planktonic bacteria. Following the removal of the planktonic cells, 2 mL of fresh LB media with the antibiotics and/or phages (10^6^ PFU/mL) were added to the biofilm-bearing wells and the plates were incubated for an additional 48 hours. The viable cell densities and phage titers in these biofilms were estimated with a procedure similar to that in [10]. In sum, following this “treatment”, the bacteria in the wells were suspended in liquid by scraping the walls and floor of the wells with a wooden applicator. To further suspend these bacteria and phage and to break-up clumps, these suspensions were passaged twice through the 29 gauge needles of 1 mL (0.33 x 12.7 mm) “insulin” syringes (Exel INT^®^). The viable cell densities of bacteria and titers of phage in these suspensions were then estimated by serial dilution and plating. The phages were titered on the lawn of ancestral *P*. *aeruginosa* PA14.

For the epithelial cell biofilm experiments, once the monolayer of epithelial cells was established as described above (between 8 and 10 days), the medium was replaced with 2 mL fresh MEM and 0.05 mL of *P*. *aeruginosa* PA14 from an overnight LB cultured diluted in MEM was inoculated to a density of approximately 10^6^ cells/well. To maintain the integrity of the epithelial cell monolayer and promote *P*. *aeruginosa* PA14 biofilm formation, the MEM used to culture human cells was supplemented with 0.4% arginine [40]. The plates were incubated at 37°C and 5% CO_2_ for 1 hour. The medium containing the unattached (planktonic) *P*. *aeruginosa* PA14 was then removed using a sterile serological pipette and replaced with fresh MEM supplemented with 10% fetal bovine serum and subsequently incubated for 8 hours. To treat the biofilms on these layers of epithelial cells, the planktonic *P*. *aeruginosa* PA14 cells were removed by aspiration, and the biofilm culture was washed twice with MEM. Fresh media containing phages, antibiotic or combination treatment was added to the wells. The presence of growing *P*. *aeruginosa* PA14 micro colonies and biofilm was assessed by microscopy with the calcofluor assay as described in [40]. The viable cell density of *P*. *aeruginosa* PA14 within the epithelial cell layer was estimated by the disruption and suspension procedure described above for the biofilms in the polystyrene plates.

### Statistical tests of synergy and facilitation

In keeping with our interest in assessing the interaction between phages and drugs in killing bacteria, we offer a formal model of the different forms and magnitudes of interaction. Let C be the cell density obtained in the control (no treatment), and let S_A_, S_B_, and S_AB_, respectively be the surviving cell density after treatment with agent A, agent B, and the combination of A and B. The fraction of cells surviving A is S_A_/C, of cells surviving B is S_B_/C, and so forth.

Facilitation requires both S_A_/C > S_AB_/C and S_B_/C > S_AB_/C. Taking logs of the first inequality,

$$\begin{array}{l} \text{log}(S_{A})-\text{log}(C)\;>\text{ log}(S_{AB})-\text{log}(C)\;\\ or\\ \text{log}(S_{AB})-\text{log}(S_{A})\;<\;0 \end{array}$$ (1)

Likewise,

$$\text{log}(S_{AB})\;-\text{ log}(S_{B})\;<0$$ (2)

The null model of no facilitation (hence no synergy either) is that the effect of combined treatment is no better than the effect of the best single treatment, so the inequalities in Eqs (1) and (2) are replaced with equalities in the test. To reject this null model, the inequality must hold for both agents. Allowing the sampling errors to be distributed normally with equal variance, the sampling distribution of each null model follows a Student’s t with degrees of freedom determined by sample sizes.

If the effects of agents A and B applied separately are equal, then requiring a 0.05 level on both A and B for rejection of the ‘no facilitation’ hypothesis would impose a type I error rate of 0.0025. In reality, the individual effects of A and B will often not be equal, in which case the fate of the null hypothesis will rest on the comparison closest to equality. We will thus conservatively apply a 0.05 criterion to both tests for rejection.

Synergy requires S_A_/C x S_B_/C > S_AB_/C. Following derivations similar to those above,

$$\text{log}(C)-\text{log}(S_{A})-\text{log}(S_{B})+\text{log}(S_{AB})<0$$ (3)

With similar assumptions as for facilitation, the sampling distribution of the left side of Eq (3) under the null model of equality follows at distribution with appropriate degrees of freedom.

## Results

Treatment efficacy was tested in several environmental contexts and for different schedules of drug and phage application. The data are typically cell counts obtained from biofilms grown for a fixed period of time (48 hours) and then treated for an additional fixed period of time (48 hours). Cell counts were obtained by destructive sampling of the biofilms and are thus limited to one count per biofilm. As such, the counts do not give information about the pharmacokinetics of the antibiotics and population and evolutionary dynamics of the bacteria and phage during treatment, and our tests of synergy and facilitation are limited to single time points.

The results sections is partitioned according to (A) simultaneous, combination treatment of ‘pure’ biofilms on polystyrene (henceforth ‘plastic’), (B) staggered, combination treatment of biofilms grown on plastic, (C) combination treatment of drug-resistant and drug-sensitive cells grown on plastic, and (D) simultaneous, combination treatment of biofilms grown on epithelial cells.

### A) Simultaneous treatment of intact biofilms grown on plastic

**Fig 3:**
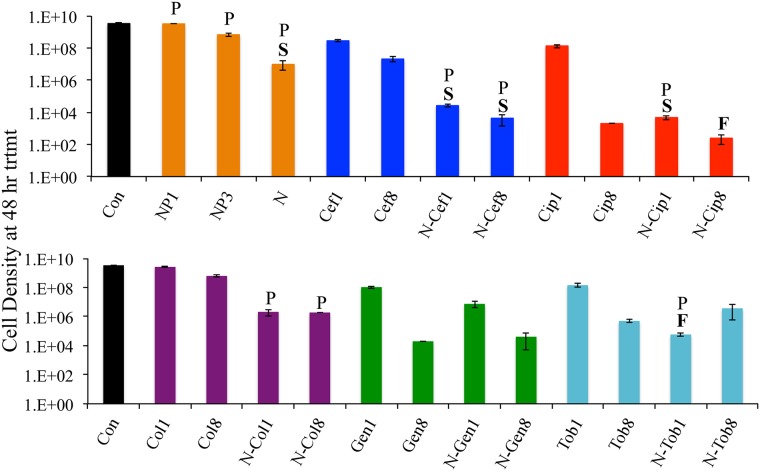
Treatment of intact biofilms grown on plastic. Viable cell densities (mean ± standard error, three replicates) in 48 hours, intact biofilm populations of *P*. *aeruginosa* PA14 on plastic then treated for 48 hours in various combinations of two phages (NP1, NP3) and/or five antibiotics (ceftazidime, ciprofloxacin, colistin, gentamicin, and tobramycin). Antibiotics were used at 1X and 8X MIC concentrations. Abbreviations are given by the first 3 letters of the drug name, and the number following the abbreviation indicates 1X or 8X MIC. A prefix N- indicates inclusion of both phages. “Con” is the untreated control with no antibiotic or phage added as treatment. A ‘P’ above the bar indicates that the phage titer at 48 hours exceeded the inoculum density by at least a factor of 10. ‘S’ indicates statistical support for synergy, ‘F’ for facilitation. Raw data of these experiments can be found in (S1 Excel File).

**Fig 4:**
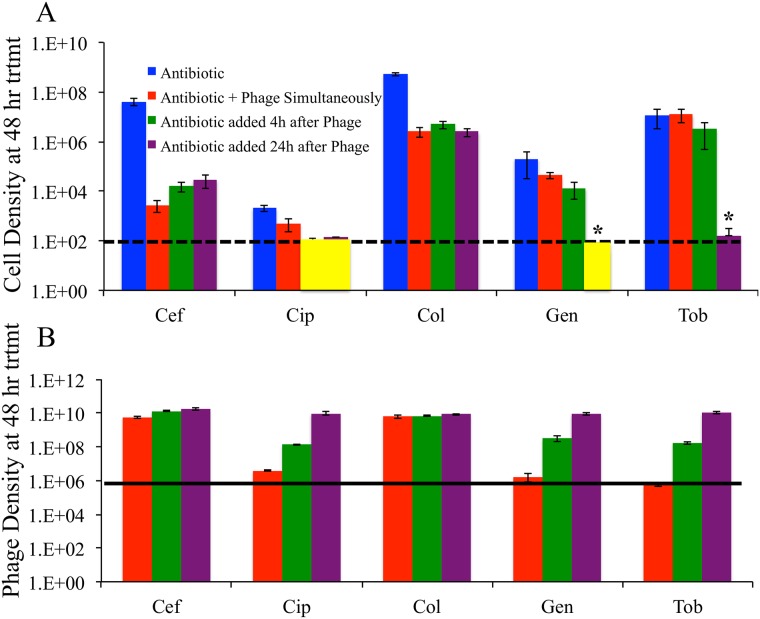
Effect of treatment order in killing *P*. *aeruginosa* PA14 on plastic. Bacteria alone were grown 48 hours, then treated with the two phages (N) for 0, 4 or 24 hours. They were then treated with the drug (8X MIC, abbreviations as in Fig 3) for the duration of treatment. Starting from the time phages were added, the culture was grown 48 hours, so the duration of treatment following antibiotic addition was shorter with the longer phage pretreatments. (**A)** Densities of viable *P*. *aeruginosa* PA14 at the end of treatment. The horizontal dashed line is the limit of detection (10^2^/mL), and yellow boxes indicate that estimates were below the limit of detection. (**B)** Densities of phage at the end of treatment. The bold black line in (B) is the initial density of phage introduced. * Indicates that the 24 hours delay of gentamycin and tobramycin each have statistically significant effects on cell density compared to simultaneous treatment (P< 0.04, when correcting for multiple comparisons; tests of significance were equivalent for a Mann-Whitney U test and a median test using a Fisher’s exact test calculation—parametric tests were not possible because of some censored data). Means and standard errors from data obtained from two independent experiments, with a combined total of 5 replicate cultures. Raw data of these experiments can be found in (S2 Excel File).

Intact biofilms were grown for 48 hours and treated for the next 48 hours (protocol of Figs 2 and 4b). Viable bacterial counts are presented in Fig 3. Some noteworthy points follow.

#### Synergy between the two phages

The two phages killed a greater fraction of cells together than expected from the product of their separate effects. This synergy may result from the fact that the two phages appear to use different receptors.

#### Synergy between phages and some drugs

The effect of phages plus drugs was synergistic for ceftazidime (at 1X and 8X MIC) and for ciprofloxacin (1X MIC only). The combination of phages plus drug was facilitative for ciprofloxacin (8X MIC) and for tobramycin (1X MIC). No synergy or facilitation was evident between phages and the other two drugs (gentamicin, colistin).

On first consideration, the results from tobramycin may seem paradoxical. When used alone, the 8X MIC of tobramycin achieved significantly greater killing than 1X MIC, which is not surprising. Yet the greatest overall killing was observed with 1X MIC tobramycin plus phages; the addition of phages to 8X MIC of this drug resulted in no greater killing than with the drug alone. This apparent paradox may stem from phage replication (amplification) being greater with 1X dose than 8X dose of tobramycin (Fig 3), which could be an effect of drug interference with phage replication at 8X MIC or an effect of the 8X MIC of the drug reducing bacteria to levels below which it can replicate [41, 42].

### B) Staggered phage and antibiotic treatment

As suggested above for tobramycin, antibiotics can be antagonistic to phage because they reduce the density of the bacteria and thus the capacity of these viruses to replicate [43]. Worse, antibiotics may even interfere with phage replication within the cell, thereby causing a reduction in phage numbers [41, 44, 45]. One way to test the effects of this possible antagonism is to treat with phage first and subsequently treat with the antibiotic, comparing the outcome with the case of simultaneous treatment. Here, we used delays of 4 and 24 hours. Results show substantial effects of delayed treatment with phage for some antibiotics but no effect for others (Fig 4). The only statistically significant effects of delay are for the 24 hours delay using gentamicin and tobramycin, but the magnitude of the effect is profound. These are two of the three drugs for which simultaneous treatment suppressed phage replication (Fig 4B). The third such drug that suppressed phage replication with simultaneous treatment (ciprofloxacin) also exhibited greater kill with phage-first treatment, but the statistics fail to reject the null hypothesis of no effect of delay. This case warrants further investigation.

### C) Phage control of antibiotic resistant bacteria

One would expect the combination of antibiotic and phage treatment to be at least somewhat effective against bacterial populations resistant to either of the single agents. One form of this hypothesis was tested—phage control of drug resistant populations [46]. Biofilm populations were established with (i) drug resistant bacteria only or (ii) a mixed population in which the majority of bacteria are susceptible to a drug and a minority are resistant to a drug. The drug-resistant population was inoculated at a density 10^−4^ relative to the sensitive cells. Notably, at the time of treatment, the density of resistant cells had already increased by as much as 10-fold. These cultures were then treated with the drug alone and with drug plus both phages, administered simultaneously. Drug levels used in these experiments were not the same used in other experiments (6X MIC was used for ciprofloxacin, 3X for gentamicin), so the effects of drugs reported here will not necessarily correspond to the effects reported in other sections of this paper. These doses were used to ensure that the drug sensitive cells would be inhibited but the drug-resistant cells would not.

**Fig 5:**
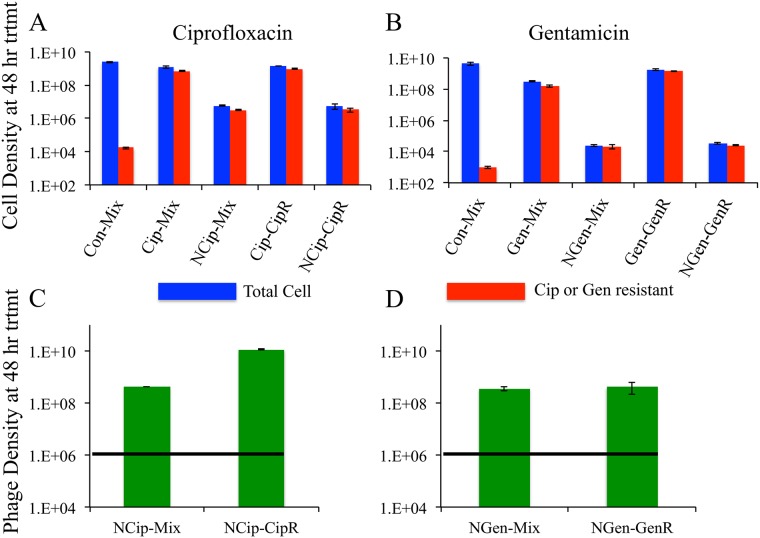
Combination of phage and antibiotic treatment on the ascent of antibiotic resistance. Treatment of *P*. *aeruginosa* PA14 biofilm populations grown for 48 hours either fully resistant to the treating drug (CipR, GenR) or containing an initial mix of susceptible and drug-resistant bacteria (designated as—Mix). Abbreviations are given by the first 3 letters of the treating drug name followed by the status of the initial population (mixed or fully drug resistant). A prefix N- indicates inclusion of both phages with the drug. **A-** Viable cell density after 48 hours of treatment with ciprofloxacin (Cip) and ciprofloxacin with phage mixture (NCip) **B-** Viable cell density after 48 hours of treatment with gentamicin (Gen) and gentamicin with phage mixture (NGen) **C-** Titers of phage in (A). **D-** Titers of phage in (B). The horizontal line in (C) and (D) is the initial density of phage. For ciprofloxacin, the differences in total cell density were not statistically significant between Cip-Mix and Cip-CipR nor between NCip-Mix and NCip-CipR. The difference in the drug resistant fraction between Con-Mix and NCip-Mix is highly significant (P << 10^−4^). For gentamicin, the differences in total cell density were not statistically significant between NGen-Mix and NGen-GenR but were statistically significant between Gen-Mix and Gen-GenR (P < 10^−4^); however the magnitude of difference in this latter case is not large. The difference in the drug resistant fraction between Con-Mix and NGen-Mix is highly significant (P < 10^−4^). Raw data of these experiments can be found in (S3 Excel File).

Several outcomes are clear (Fig 5):

1. Perhaps the most striking result is that regardless of whether the treatment was drug alone or drug plus phage, the evolution of resistant cells was virtually the same. The bacteria that survived treatment were nearly all resistant, and the density of surviving bacteria was approximately the same whether the inoculum had a minority population of resistant cells or was entirely resistant.
2. When the inoculum had only a minority of drug-resistant cells, treatment with drugs alone allowed bacterial densities to nearly reach the untreated/control levels due to complete takeover by the resistant fraction.
3. Combination treatment with phages (and drugs) did prevent bacterial densities from reaching control levels. The bacterial densities were suppressed 3 and 5 orders of magnitude compared to controls. This suppression is nearly equal to that of the drug-only treatments in the absence of resistance.
4. The phages did not suppress resistant bacterial densities to the level expected if resistant cells were being killed at the same rate as sensitive cells (e.g., the rate in Fig 3). For both drugs, the densities of resistant cells surviving combined treatment were significantly higher than densities of resistant cells in the control. It thus appears that the resistant cells grew somewhat under combined treatment.

Point (iv) underscores the fact that we do not know whether surviving bacterial numbers are static or dynamic. A strict static interpretation would mean that following a short period of treatment the number of surviving cells descends to a level at which it remains thereafter with no further cell loss or reproduction. A dynamic interpretation would allow for the number of surviving cells to be changing over time and for that number to result from a balance between ongoing bacterial death and reproduction. The fact that drug-resistant cell numbers increased during combined treatment suggests a dynamic interpretation, although the potential complexity of this process otherwise lies well beyond discovery from the methods used here.

### D) Treating biofilms on cultured nasopharyngeal cells yield similar results

As the treatment of biofilms grown on plastic may not give the same results as biofilms in patients, we explored the treatment of biofilms grown on human cells. *P*. *aeruginosa* PA14 was added to confluent monolayers of human nasopharyngeal cells and allowed to grow and establish biofilms for 8 hours. A longer period for growth was avoided as *P*. *aeruginosa* PA14 killed epithelial cells when allowed to grow for durations exceeding 8 hours. For this same reason, we sampled the treated cultures 12 hours after treatment rather than 48 hours afterward as we had done in the prior experiments with biofilms on plastic.

**Fig 6:**
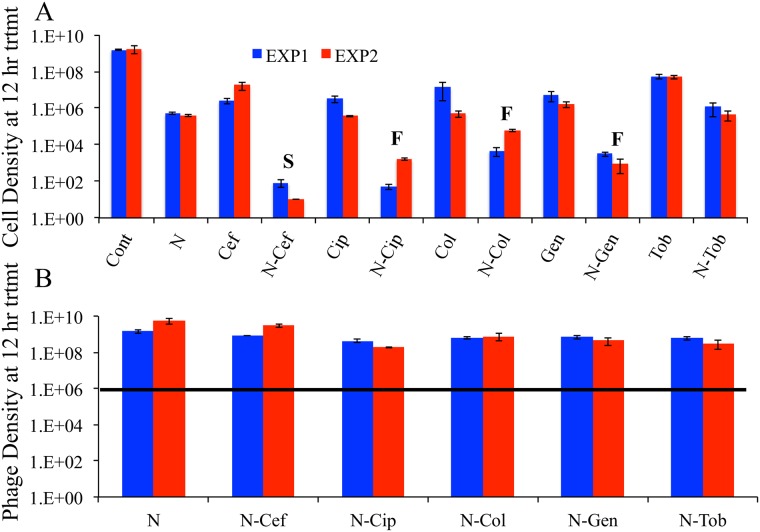
Treatment of biofilm grown on epithelial cells. Treatment of 8 hours old biofilm population of *P*. *aeruginosa* PA14 on human epithelial cells treated with a mixture of NP1 and NP3 phage (N) and 1X MIC concentrations of ceftazidime, ciprofloxacin, colistin, gentamicin and tobramycin. **A-** Viable cell densities of *Pseudomonas* estimated in two independent experiments (red, blue) at 12 hours of exposure to the treatments. ‘S’ indicates statistical support for synergy and ‘F’ for facilitation. **B-** Densities of phage in two independent experiments sampled 12 hours after treatment. The bold black line in B is the density of the mixture of NP1 and NP3 introduced to the biofilm. Mean ± standard error for 3 replicates. Raw data of these experiments can be found in (S4 Excel File).

Treatment with all 5 antibiotics alone prevented the growth of the bacteria, relative to the untreated biofilm control (Fig 6). Save for tobramycin, simultaneous treatment with the phage and antibiotics markedly improved the efficacy of these drugs in killing the bacteria (Fig 6A).

In all cases, the phages were able to replicate (Fig 6B). For most drugs, phage replication could be explained by the use of a 1X MIC drug concentration, as seen in Fig 3. However, 1X MIC gentamycin inhibited phage replication when cells were grown on plastic (Fig 3), so the fact that phage replicated on bacteria grown on epithelial cells with 1X MIC indicates an effect of media or epithelial cells in permitting phage replication.

## Discussion

In response to the increasing frequencies of pathogens resistant to multiple antibiotics, there has been a resurgence of interest in theoretical and experimental studies exploring the efficacy of bacteriophage in combination with antibiotics to treat bacterial infections [14–16, 28, 47]. Taken at large, the results of these studies support the proposition that phage can increase the efficacy of antibiotics to treat these infections and facilitate the breaking up the of biofilms that commonly thwart antibiotic therapy [30]. In addition to this pharmacodynamics synergy, there is evidence for evolutionary synergy by phage treatment increasing the susceptibility to antibiotics [31]. We interpret the results of our study as additional evidence for both pharmacodynamic synergy of phage and bactericidal antibiotics in treating biofilm populations of *P*. *aeruginosa* PA14 and the prevention of treatment failure due to the ascent of minority populations resistant to the treating antibiotic.

There are six results of particular relevance to the clinical potential of combination phage and antibiotic therapy.

1. As measured by the extent to which bacteria in biofilms are killed, using pairs of phages with an antibiotic can be more effective than using the antibiotic alone. Stated another way, the interaction between these phages and drugs can be synergistic, sometimes profoundly so.
2. The combination of drugs and phages can kill more bacteria in biofilms than either agent alone. In some cases, the combination kills more bacteria than would be expected if the agents were acting independently.
3. When combined with phage, some antibiotics may be more effective at lower doses than higher.
4. The efficacy of some antibiotics for treating biofilm infections can be considerably augmented when the phage are administered before the antibiotic rather than if they are simultaneously administered [14, 15].
5. Phage can prevent treatment failure due to the ascent to high densities by minority populations of bacteria resistant to the treating antibiotic [15, 31, 46].
6. Save for tobramycin, phages are effective as an adjunct to antibiotics when the biofilms are formed on layers of human nasopharyngeal epithelial cells

In this study, we focused on the potential practical application of combinations of antibiotics and phage for treating biofilm infection. We have not explored the pharmaco—population and evolutionary dynamic processes responsible for the observed results. Elucidating these processes, perhaps with the aid of mathematical and computer simulation models, is certainly needed to understand and predict the conditions under which combinations of antibiotics and phage will be more effective than antibiotics alone.

### Implications for the clinical use of combining of antibiotic and phage therapy

This study is limited as we restricted our work to *in vitro* experiments. The successful treatment of experimental infections with laboratory animals e.g. [30], will doubtless add credence to purely *in vitro* studies. It is clear, however, that without successful trials in humans, phage therapy in any form will not be implemented in the United States or most other countries. We suggest rather than the treatment of systemic infections, combined antibiotic and phage treatment of skin infections, like the ulcers of diabetics, may well be an acceptable way to initiate a clinical evaluation of the efficacy of phage as an adjunct to antibiotics for the treatment of human infections. Phages abound in our environment and are almost certainly not going to have toxic or otherwise deleterious effects in humans, especially when administered topically. Unlike ‘phage only’ therapy, it will be possible to perform clinical trials to evaluate the efficacy of phage as adjuncts to the established antibiotic therapy and perform "non-inferiority" trials [48].

## Supporting Information

S1 Excel File (XLSX)

S2 Excel File (XLSX)

S3 Excel File (XLSX)

S4 Excel File (XLSX)

S1 Fig Phage Morphology. Transmission electron micrograph of negatively stained (**A**) NP1 and (**B**) NP3 phage, bar of 50 nm.
(TIF)

S2 Fig Genome annotation of NP1 and NP3 bacteriophage. Predicted ORFs were plotted by using the Artemis DNA plotter. Direction of the arrows on the map indicates orientation of the genes; PKP, 3'-phosphatase, 5'-polynucleotide kinase; TSX, Thymidylate synthase thyX. ORF for structural genes indicated as green, lysis in yellow, and DNA metabolism ORF as red box. The innermost purple-green ring shows GC skew, whereas the purple-green ring in the middle shows GC content (outer and inner peaks indicating above or below average GC content, respectively).
(TIF)

S1 Text File Phage morphology and genome sequencing. (DOCX)
