# Supplementary figures and images for: Synergy and Order Effects of Antibiotics and Phages in Killing Pseudomonas aeruginosa Biofilms
Source: PLoS One. 2017 Jan 11;12(1):e0168615. doi: 10.1371/journal.pone.0168615 (PMC5226664; doi:10.1371/journal.pone.0168615)

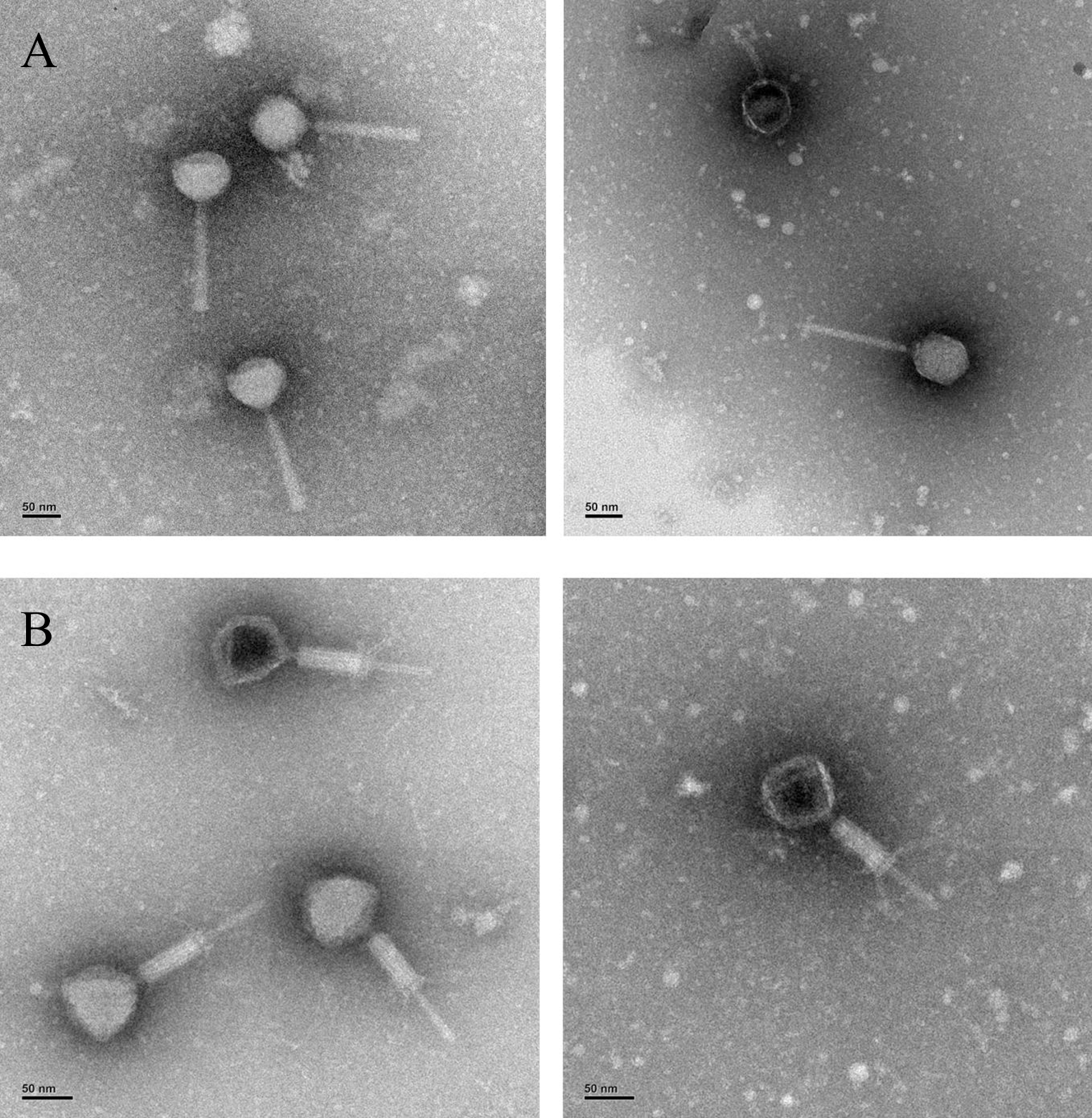

Supplement: S1 Fig — Transmission electron micrograph of negatively stained (A) NP1 and (B) NP3 phage, bar of 50 nm. (TIF) [file pone.0168615.s005.tif]

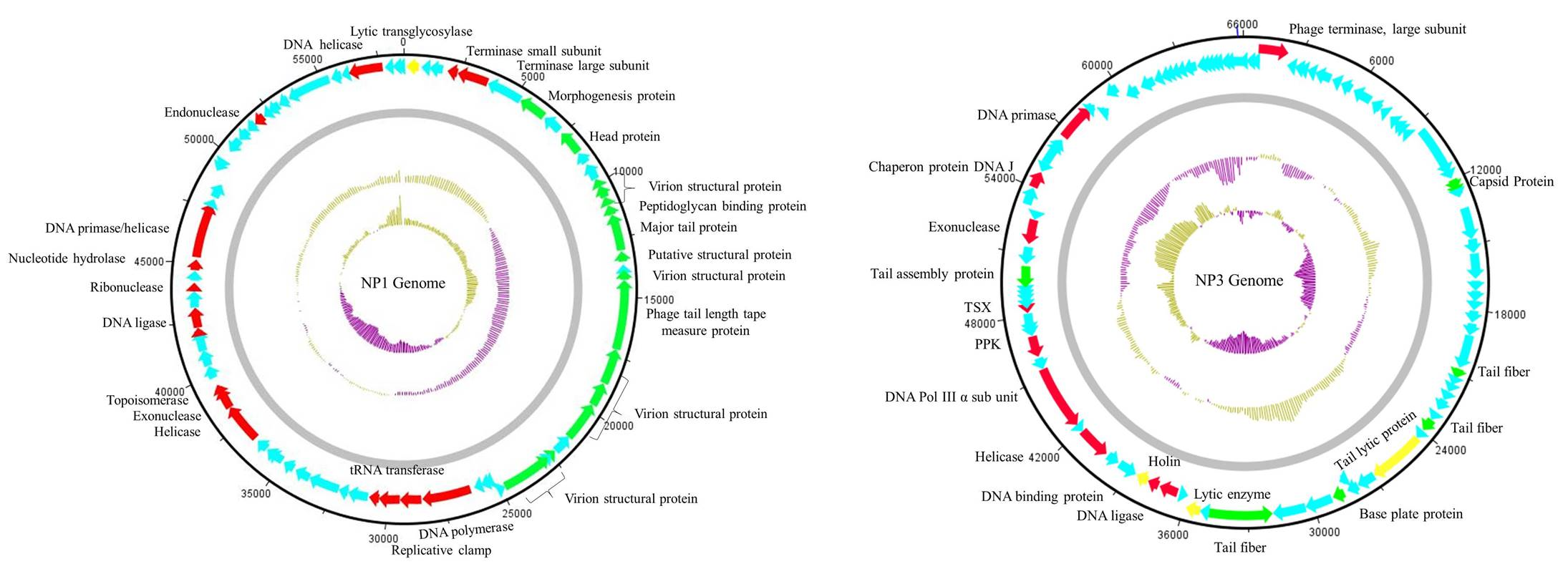

Supplement: S2 Fig — Predicted ORFs were plotted by using the Artemis DNA plotter. Direction of the arrows on the map indicates orientation of the genes; PKP, 3'-phosphatase, 5'-polynucleotide kinase; TSX, Thymidylate synthase thyX. ORF for structural genes indicated as green, lysis in yellow, and DNA metabolism ORF as red box. The innermost purple-green ring shows GC skew, whereas the purple-green ring in the middle shows GC content (outer and inner peaks indicating above or below average GC content, respectively). (TIF) [file pone.0168615.s006.tif]
