# Supplementary material for: Synergy and Order Effects of Antibiotics and Phages in Killing Pseudomonas aeruginosa Biofilms
Source: PLoS One. 2017 Jan 11;12(1):e0168615. doi: 10.1371/journal.pone.0168615 (PMC5226664; doi:10.1371/journal.pone.0168615)
Supplement: S1 Text File — (DOCX) [file pone.0168615.s007.docx]

**Phage morphology and genome sequencing**

**Phage morphology**

Both NP1 and NP3 were negatively stained with 1% phosphotunstic acid (PTA) on 400 mesh carbon film coated copper grids and examined with a JOEL JEM-1400 transmission electron microscope (Tokyo, Japan) operated at 120 kV. Images were taken with a Gatan 2k x 2k US1000 CCD camera (Gatan Inc., Pleasanton, CA) at the Robert P. Apkarian Integrated Electron Microscopy Core facility, Emory Atlanta. Based on their morphological features, the NP phages were classified according to the guidelines of the International Committee on Taxonomy of Viruses.

The transmission electron micrograph revealed phage NP1 has an icosahedral head (length 80 nm, width 56 nm) and flexible tail (137 nm), and was classified under family *siphoviridae*. While phage NP3 has an icosahedral head (length of 77 nm, width 75 nm) and contractile tail, which comprises of a neck, a contractile sheath, and a central tube (length 146 nm). It was classified under family *myoviridae* (S1 Fig).

**Genome sequencing**

For the genome isolation, the NP1 and NP3 phages were grown on *P. aeruginosa* PA14. The phages in the lysate were precipitated with 10% PEG 8000.  The pellet was re-suspended in 10 mM tris-HCl, pH 7.6, and 10 mM MgCl2. The phages were purified by equilibrium density centrifugation in CsCl. The phage DNA was isolated by phenol extraction and submitted to the core facility for next-generation sequencing on the Illumina MiSeq platform, where a mate-pair sequencing library was created before subjected to sequencing.  Raw sequence data were assembled using the CLC Bio v8.5 Genomics Workbench program (CLC Inc., Aarhus, Denmark). DNA sequencing was performed at the University of Texas at Austin, Institute for Cellular and Molecular Biology Genomic Sequencing and Analysis Facility, US.

NP1 and NP3 Genome consist of 58566 and 66063 base pairs with an overall 58.39 and 54.41% of GC content, respectively. Genome examination revealed NP1 contain 74 protein coding genes and NP3 to have 90 protein coding genes (S2 Fig). Among these coding genes, 57 % and 26 % genes have been identified to share significant sequence similarities to the genes with known function in NP1 and NP3, respectively. No bacterial virulence or antibiotic resistance genes were detected in NP1 and NP3 genomes.
